# Hospital and Institutional News

**Published:** 1921-01-29

**Authors:** 


### HOSPITAL AND INSTITUTIONAL NEWS.

### THE MEDICAL SERVICE FOR SCOTLAND.

The memorandum and letter which have been circulated by the Medical Guild in Scotland, with reference to the interim report of the Consultative Council on Medical and Allied Services, and under the title "A State Socialistic Scheme of Medical Service for Scotland," seems to be a particularly virulent and probably undeserved criticism of a set of most well-intentioned resolutions. As we understand the Guild's attitude, it condemns indiscriminately any form of State medical supervision, and sees in the Scottish report nothing but selection of relations between the community and the medical profession most favourable for getting the thin end of the wedge of State Socialism into the country. The importance of the "family doctor" is specifically urged in the report. According to the Guild, however, it is at the same time implied that "the family doctor is to be lowered to the status of a scullery-maid in a State medical kitchen." We agree that it will materially help matters if all members of the profession will co-operate and instruct their patients as to the true character of this and other proposed schemes, but hardly, we think, if such extremist views of the situation be taken. It is fully understandable that a certain number of medical men in a border-line grade of private practice will not welcome an upward extension of State supervision in matters medical. But much as we appreciate the possible disadvantage to this professional few, we also realise that extension and improvement of organised medical care for the border-line public is a matter of national urgency. If the State is not in any circumstances to provide it, perhaps the Guild will also indicate to the medical profession the means by which they can themselves solve the problem. Surely they do not suggest that the situation is satisfactory as it stands, and so good that it should be left entirely alone.

### THE MANCHESTER HOSPITAL CO-OPERATION SCHEME.

Our readers will appreciate fuller details of the co-operation arranged between the Guardians and the voluntary hospitals in Manchester. The Guardians of the Manchester Union have recently adopted a scheme for separate accommodation in their hospitals at Crumpsall and Withington of adult persons in the Manchester area suffering from acute but not infectious complaints who cannot secure early admission into the voluntary hospitals. The Guardians are anxious to do their utmost to relieve the pressure on the voluntary hospitals, hence their desire to utilise the limited amount of accommodation they have available for middle-class patients. The portions of the buildings to be used will be known as the Auxiliary Hospital, Crescent Road, and the Auxiliary Hospital, Nell Lane, respectively, and it is proposed to admit patients thereto as from February 1 at a charge of three guineas per week, inclusive. It is expected that this amount will entirely cover the cost of maintenance and treatment of the patients, and in consequence every effort has been made to deal with them on the lines of private patients. Each of the hospitals referred to has, besides the usual operating-theatre and special departments, an *x*-ray apparatus and appliances for electrical treatment. Massage treatment is also given. The medical staff of the hospitals includes such visiting specialists as physicians, surgeons, a gynæcologist, an aurist and laryngologist, an ophthalmic surgeon, and radiologists. An efficient nursing staff is also employed. Application for admission to these hospitals should be made to the medical officer or matron at the hospital. The scheme will not, except to a minor extent, reduce the beds available for the sick poor, and any person unable to pay the charge above mentioned can still receive treatment in the Guardians' hospitals. The Guardians are willing to permit medical practitioners (including consultants) to visit their patients in the hospitals and consult with the Guardians' medical staff if they desire to do so.

### EVERYDAY MEDICINE.

On page 405 of our present issue we give the first of a series of medical articles written essentially from the standpoint of practical medicine and confined entirely to application of first principles in solution of constantly recurring problems in everyday practice. Our subjects have been chosen after discussion with consultants well qualified to judge of the points most often presenting difficulty to the busy general practitioner, and although to the more experienced of our professional readers we may present nothing new, yet we hope that to some, these notes will have that value attaching to a straightforward exposition of the essential, and only the essential, points in oft-debated problems.

### THE SUSSEX EXPERIMENT.

We are glad to see that the lay press has understood and correspondingly praised the useful and highly interesting experiment inaugurated by Dr. Gordon Dill in his medical service scheme for Sussex. Even from the super-critical quarters we read that "here is a way out of the official morass." In any case, the great point is that such a scheme as this helps directly and indirectly in maintenance of the majority of the voluntary system's ideals and that without the preponderant menace of more and yet more rates and taxes. Also it is decidedly an instance where the medical profession, by getting together in the public cause, will improve their own position in local politics.

### PHYSIOLOGICAL RESEARCH.

As a sequence to the threatened withdrawal from the physiological laboratory at South Kensington of monetary support by the L.C.C. comes the report that the Senate of the University desires the laboratory to move from its present quarters at the end of July next. According to Professor Halliburton, this action must arise indirectly from a misunderstanding on the part of the L.C.C., for although the Physiological Laboratory relinquished some of its space during the war, the departure was understood as temporary only, and in no sense implied an intended break-up of this research department. With the writer, we trust that the L.C.C., once the true position is made clear to them, will agree to continue their annual £500. The suggestion, also, that the space at South Kensington is required for clerical work seems singularly ill-advised as, on the face of it, the problem of finding space for these clerks elsewhere seems far simpler than that of re-erecting a lot of complicated laboratory equipment. The recent letter in *The Times*, signed by many now well-known scientists, all of whom have in the past worked in these laboratories, should clearly bring home the urgency of the situation. At South Kensington the Institution holds a unique position in providing circumstances where neither the necessities of elementary teaching nor financial interests can clash with the interests of research in the true meaning of the word. We sincerely hope to see a reconsideration of these reported decisions.

### A DATUM LINE FOR PATIENTS.

With a deficit of £350 and a sum of £5,000 still to raise to meet the £15,000 incurred on renovation, the Archdeacon of Cornwall has offered a suggestion, from the chair, to the governors of the Royal Cornwall Infirmary at Truro. Every effort, he told the meeting over which he lately presided, must be made to maintain the voluntary system. There are two means of doing so. One is a system of special contributions from firms. The other to follow the example set by the Exeter Hospital and to make a rule not to receive into the hospital, except by special permission of the committee, any person earning more than £3 a week. The maximum charge is 10s., but the scale varies according to the ability of the patient to pay. A long discussion followed. The upshot was that every patient, not necessitous, should contribute during treatment towards the cost of maintenance. The committee was therefore instructed to form a scheme to make the resolution effective. We take this to mean that the Exeter plan mentioned will be followed, and that what it is the fashion to call a datum line will be drawn, above which no patient will be treated free of charge. It may be said that this is only another way of assessing ability to pay, but the voluntary system is elastic, and the different applications of a generally-favoured scheme are always of interest because of the different needs of different districts.

### AN EARLY REPORT.

Mr. Edward Hemingway, the Secretary of the General Infirmary, Dewsbury, is naturally proud of producing, as early as January 11, the first proof of the audited accounts for 1920. He has every cause to be proud, and we congratulate him and inquire whether this feat has been beaten elsewhere. But we should have been better pleased if the accounts had been strictly in accordance with the uniform system, for it is plainly more simple to produce a statement of cash received and cash paid than a proper income and expenditure account for the year—*i.e.*, one including all income due and expenditure incurred belonging to the year, even though the cash transactions did not take place within the year. But an important point is that the Dewsbury Infirmary shows an excellent financial statement, the position at the end of 1920 being better than at the close of the preceding year, and the endowment fund having been augmented by £1,500.

### HOSPITAL FIRE AT ALTERYN.

A wooden pavilion, fortunately unoccupied, was lately destroyed by fire in the grounds of Alteryn Fever Hospital, Newport. The fire was seen to issue from the pavilion kitchen, and the matron, Miss F. Simpson, gave the alarm. Though the building was destroyed, the brigade was able to save the portion in which a large amount of equipment was stored. Dr. J. Howard Jones, the medical superintendent, was quickly on the scene, and doctors from the town, and two motor-ambulances from Newport also attended. The damage is estimated at £1,000. It is noteworthy that the fire started in the kitchen, where for the first time for a long period a fire had been lit. None of the hospital patients were disturbed, but a large crowd assembled, since the hospital stands on a hill, from which the flames were visible at a great distance.

### MEMORIAL TO BART'S MEN.

Mr. Girling Ball has invited expressions of opinion as to what form should be taken by the proposed memorial to St. Bartholomew's men who lost their lives in the war. Mr. Ball does not himself like the tablet form. He says the Martyrs' Memorial is daily passed; besides, it is not wished that Bart's men, who went gladly and met their fate joyously, should be thought of as martyrs. Several suggestions have come in—a brass in the library, a stone obelisk in the square, and a memorial in the surgery. The last idea is Mr. Girling Ball's own, and seems excellent, as the monument could not fail to be seen by, and teach its lesson to, a great many people in the course of the year. We understand that the funds to defray the cost have not yet been raised, but this should present no difficulty, as there are many thousands of Bart's men the world over who would wish to contribute.

### TWO IMPORTANT PROVINCIAL GIFTS.

At a recent meeting of the Board of King Edward VII. Hospital, Windsor, the welcome announcement was made that Mr. Dhunjibhoy Bomanji, an Indian gentleman of Bombay, now of The Willows, Windsor, has presented £5,000 to the institution. The gift is free of any conditions and the object of Mr. Bomanji is to help the splendid work the hospital is doing for the benefit of the sick and suffering. The Board trust that this gift will stimulate others. Large manufacturing concerns are being developed at Slough, and it is hoped that the promoters will recognise their responsibilities to the hospital. Another notable gift, an anonymous one, is Whitehaven Castle and £20,000 towards the cost of altering the building for the purposes of a hospital, which was announced at a meeting of the Governors of Whitehaven Infirmary as having been accepted. A further sum of £10,000 will be required, and towards this Mr. James McGowan gave £1,000 before the meeting closed. The hospital is to take the form of a war memorial.

### THE SELECTION OF STUDENTS.

THE January number of the *Edinburgh Medical Journal* publishes in full, together with a commentary, Dr. Norman Walker's report to the Scottish Royal Colleges upon the machinery of medical education in the United States and their recently instituted National Board of Medical Examiners. Without entering into the international bearing of this latter development, it is interesting to realise the remarkably thorough manner in which selection is made among the excessive number of students applying to enter certain American medical schools. It is not ours to detail the ideal to be sought in the embryo doctor, but it may be mentioned that, in the quoted instance, the applicants' numbers were halved by strict scrutiny of their educational qualifications. A schedule of inquiry had been sent to all those concerned with the candidates' previous educations and a report obtained upon personal appearance, manner with associates, initiative, ambition, industry, tact, and the use of oral and written English. Finally, the numbers were still further reduced by mental tests and a series of examinations on the Binet method. Even at the end of the first year of the medical curriculum unsuitable students were again weeded out. The whole sounds a drastic process, but, undoubtedly, the schools and the profession in this country have every right to select those most likely to benefit by educational facilities provided and could use their powers with ample tact and discretion. Consideration of the existing American customs is not a little interesting, in view of the recent decision at University College Hospital to limit the number of women students.

### MEDICAL SCHOLARSHIPS.

IN connection with the possibility of the selective process developing in the near future, it is to be remembered that there are a considerable number of medical scholarships open annually for competition. Those most recently announced by the London Inter-Collegiate Scholarships Board are seventeen, with an aggregate value of about £1,580, tenable in the Faculty of Medical Sciences of University College and King's College and in the medical schools of Westminster, King's College, University College, the Royal Free, and the London Hospitals. They will be offered for competition on June 28, and full particulars are obtainable from the Secretary to the Board, S. C. Ranner, Esq., M.A., at King's College Hospital Medical School.

### CARDIFF CORPORATION'S GENEROSITY.

WE were glad to see that the finance committee of the Cardiff Corporation has agreed to increase its (annual) donation to King Edward VII. Hospital from £210 to £500. The matter will be again considered at the end of the year. Some of the employees of the Corporation already contribute, and a monthly cheque is received from them by the hospital. Mr. Leonard Rea, the superintendent, will be gratified that the letter which he addressed to the Corporation has been so generously received. We hope that the Corporation's example will be followed by other public bodies in the neighbourhood, and that the Corporation's employees will all fall into line, and agree to a weekly contribution of 2d. from every one of their number.

### £100,000 RAISED BY AN APPEAL.

WHAT can be accomplished in the raising of money for a popular hospital in a comparatively short time when there is a man of energy behind it is demonstrated by the result of Mr. Peter Wright's appeal on behalf of the Royal Gwent Hospital at Newport. A sum of £43,332 is already in hand, while there are promises which may be relied upon of an additional £51,285, making a grand total up to date of £94,617. All this has been done, it should be remarked, on an expenditure of only £900. When we survey the incidents of the campaign, three facts stand out: the power of a personality behind such a movement; the value of energy and novelty in prosecuting it; and, thirdly, the generous way in which the working class, through their trade unions, can furnish hospitals with the wherewithal if they are won over by tactful treatment. Newport's fund is for the extension of the Royal Gwent Hospital, and was launched last year by Mr. Peter Wright when Mayor of the town.

### PAYING PATIENTS AT THE ROYAL FREE.

THE wards at the Royal Free Hospital for women patients paying the whole cost of treatment will shortly be opened. They are part of an extension of ward accommodation which is awaiting the provision of the necessary funds. The aim is to provide 100 beds for all classes of paying patients who will contribute their cost of maintenance; the present wards are more particularly for the treatment of diseases peculiar to women. The method at present is to advise patients waiting admission that their name has been registered for admission, and that they will be admitted as soon as a bed is available; and to ask them what sum they can contribute towards the cost of maintenance, which in 1919 amounted to £3 8s. per week. This method of collection and registration has not materially added to the cost of maintenance, and it is found that patients gladly avail themselves of the opportunity and contribute to the full extent of their ability.

### A GREAT FINANCIAL FEAT.

THE Establishment Governors of the North Staffordshire Infirmary have contributed the record sum, through the establishments, of £17,337. There is, alas, still a deficiency on the income of the Infirmary of £6,800. Dr. John Russell has presented, in this connection, an historic document, which records details of an annual meeting of the governors of the Infirmary on November 17, 1820. Mr. W. Yates was in the chair, when the establishment subscriptions amounted to £275. This was in the days when the institution, much smaller than it has since become, was housed in Etruria Vale. The rise of £3,500 in the contributions from establishments appears reflected in every item of the receipts. The total income was £51,129 compared with £44,745. But, among other expenses, rent, rates, and taxes have risen from £360 to £670! It was decided that deputations should visit works not already contributing, that contributions from the workers should be uniform, that the Pottery Managers' and Officials' Association should be invited to help, and that insurance companies should be approached for subscriptions. Now that the Mayor's Memorial Fund has been closed the Infirmary governors are to launch an appeal of their own.

### THE NEED OF THE CHILDREN.

The East London Hospital for Children have appointed an organising secretary in the appeal department, and this lady, who signs herself Janie Sargon, and knows how to write a good and convincing letter, has, in defiance of Sir Ernest Hatch's claim that there is an appeal armistice now on, quickly got to work. Our sympathies are aroused first of all by the reminder that the East London Children's hospital was started at his own expense, by a young doctor, Nathaniel Heckford, and his wife, who gave their whole means—and he his life—for the sake of the suffering poor. Then we come to plain everyday facts—an expenditure of £22,000, an assured income of £10,000, and a debt to the bank of £9,000. The present position is trying enough, but the future must be thought of as well, and the selling of stock, at present values, does very little to help and greatly embarrasses the future of the hospital. The capital must therefore be strengthened if the well-being of future generations is accepted as a present-day responsibility and not only present gifts, especially annual subscriptions, but legacies are appealed for.

### A BIRMINGHAM EXPERIMENT.

An interesting experiment is being made at the Birmingham Children's Hospital, which proposes to admit private patients. In the building in Ladywood Road about 100 cots are vacant because of the hospital's financial difficulties. Therefore a private ward of six cots is to be opened for paying patients, at three guineas a week, exclusive of medical treatment, the cost of which will be arranged between the medical staff and the patients' relatives or friends, subject to a maximum fee of three guineas a week, or £21, irrespective of the patients' length of stay in the hospital. The children's hospitals generally have not favoured weekly contributions from patients, a system which, perhaps, is more popular in London than in the provinces. It is noteworthy that Mr. Harold Shrimpton, the House Governor, has urged the need of closer co-operation and a central organisation for hospitals, apparently on the lines recommended by Sir Napier Burnett, to whose proposal we lately referred.

### MUNICIPAL CONTRIBUTIONS FOR MATERNITY CASES.

In a letter to the Westminster City Council on behalf of Queen Charlotte's Lying-in Hospital, it is stated that the St. Pancras and the Hampstead Borough Councils pay to the hospital twenty shillings for every patient resident in the boroughs admitted to the hospital for confinement, and the hospital in return issues letters of recommendation to enable the Councils to recommend patients for admission, one letter being issued for every £8 contributed, which was the estimated average cost per patient during 1920. It is also understood that the St. Pancras Borough Council also makes capitation grants in respect of midwifery services provided in the patients' homes at the rate of 5s. a case. It is pointed out that the number of women received into the hospital from Westminster each year is between 50 and 100, and the secretary of the hospital inquired if the City Council would be prepared to make an arrangement on the lines indicated. The Health Committee of the City Council, however, reports that it understands that in no case is a patient received into the hospital without the production of a subscriber's letter, and in these circumstances it does not consider that the Council should also subscribe for patients received into the hospital from addresses in Westminster.

### AN ARMY DENTAL CORPS.

Those of our readers who experienced during the war the often extraordinary difficulty in obtaining skilled dental attention for the men under their care will appreciate the advent of an Army Dental Corps. A Royal Warrant has been issued to authorise its formation, and the corps will be administered by the Director-General, A.M.S., and will be a joint service for the Army and the R.A.F. Commissions as lieutenants may be given to persons duly qualified under regulations approved by the Army Council, and the *personnel* will be required to serve under either force and will be interchangeable.

### THIS WEEK'S DRUG MARKET.

A slight improvement in the tone of business is discernible, but it is only very slight. There is, however, a rather better inquiry, which may lead to actual orders in due course. The downward tendency of prices is nevertheless still in evidence. At the recent public sale of drugs very large quantities were offered, but the bidding was extremely slow, and an extraordinary large proportion of the offerings failed to find buyers. Ipecacuanha was lower in price, as also were gum benzoin and Jamaica sarsaparilla, but as so few purchases were made it is difficult to fix prices; in any case, the only way to have effected sales would have been to make price concessions. In synthetic drugs there is a slightly steadier tone, although here and there cheap parcels are offered.

